# Two bullets in the gun: combining immunotherapy with chemotherapy to defeat neuroblastoma by targeting adrenergic-mesenchymal plasticity

**DOI:** 10.3389/fimmu.2023.1268645

**Published:** 2023-10-02

**Authors:** Silvia D’Amico, Patrizia Tempora, Paula Gragera, Kamila Król, Ombretta Melaiu, Maria Antonietta De Ioris, Franco Locatelli, Doriana Fruci

**Affiliations:** ^1^ Department of Paediatric Haematology/Oncology and Cell and Gene Therapy, Bambino Gesù Children Hospital, Istituto di Ricovero e Cura a Carattere Scientifico (IRCCS), Rome, Italy; ^2^ Department of Clinical Sciences and Translational Medicine, University of Rome “Tor Vergata”, Rome, Italy; ^3^ Department of Pediatrics, Catholic University of the Sacred Heart, Rome, Italy

**Keywords:** neuroblastoma, tumor microenvironment, adrenergic to mesenchymal transition, tumor plasticity, drug resistance, metronomic chemotherapy, immunotherapy

## Abstract

Neuroblastoma (NB) is a childhood tumor that originates in the peripheral sympathetic nervous system and is responsible for 15% of cancer-related deaths in the pediatric population. Despite intensive multimodal treatment, many patients with high-risk NB relapse and develop a therapy-resistant tumor. One of the phenomena related to therapeutic resistance is intratumor heterogeneity resulting from the adaptation of tumor cells in response to different selective environmental pressures. The transcriptional and epigenetic profiling of NB tissue has recently revealed the existence of two distinct cellular identities in the NB, termed adrenergic (ADRN) and mesenchymal (MES), which can spontaneously interconvert through epigenetic regulation. This phenomenon, known as tumor plasticity, has a major impact on cancer pathogenesis. The aim of this review is to describe the peculiarities of these two cell states, and how their plasticity affects the response to current therapeutic treatments, with special focus on the immunogenic potential of MES cells. Furthermore, we will discuss the opportunity to combine immunotherapy with chemotherapy to counteract NB phenotypic interconversion.

## 1 Introduction

Neuroblastoma (NB) is a malignant tumor arising from primitive neuronal crest cells of the developing sympathetic nervous system (SNS), and is the most common extracranial solid tumor in children, responsible for 15% of childhood cancer deaths (1, 2). Patients with high-risk NB receive a very intensive multimodal treatment regime, including induction chemotherapy, surgery, high-dose treatment with allogeneic stem cell transplantation and radiotherapy. This is then followed by isotretinoin and anti-GD2 monoclonal antibody to treat any residual disease (3). Although most high-risk NB patients initially respond to treatment, often with complete clinical remission, many of these relapse by developing therapy-resistant tumors (4). The onset of chemoresistance is a phenomenon typically related to intratumoral heterogeneity (ITH) (4).

NB ITH originally demonstrated in isogenic tumor-derived cell lines consisted on the presence of multiple cell types that differed in morphology, tumorigenic properties, and biochemical markers (5, 6). Based on their characteristics, the cells were defined as: (i) neuroblastic (type N), (ii) substrate-adherent (type S) and (iii) intermediate (type I) with a mixed and more aggressive phenotype, referred to as ‘malignant NB stem cells’ (5, 6).

Recent gene expression and epigenetic profiling of 33 different NB cell lines have provided insight into the details of NB ITH. van Groningen and colleagues identified two predominant cell identities, named adrenergic (ADRN) and mesenchymal (MES), that can spontaneously interconvert through epigenetic regulation (7–9). The phenotypic characteristics of these two cell populations are largely determined by the activation of specific transcriptional circuitries: while ADRN cells express markers of sympatho-adrenergic differentiation, MES cells appear undifferentiated and more similar to their neural crest progenitors (7, 8). Interestingly, the ADRN and MES classification coincides with the characteristics previously described for N/I-type and S-type cells, respectively (10).

The potential for interconversion between different cell states, is known as tumour plasticity and has recently been proposed as a new ‘emerging hallmark of cancer’ (11). Indeed, it can lead to the expression of phenotypic characteristics that may be advantageous in the presence of selective pressures and contribute to drug resistance and cancer progression.

In this review, we focus on the peculiarities of ADRN and MES cell states, and how their plasticity may influence the response to current therapeutic standards. Furthermore, we discuss how the combination of chemotherapy and immunotherapy has the potential to successfully target the dual nature of NB, possibly improving the prognosis of these young patients.

## 2 Plasticity of neuroblastoma: from adrenergic to mesenchymal cell lineage and back

**Figure 1:**
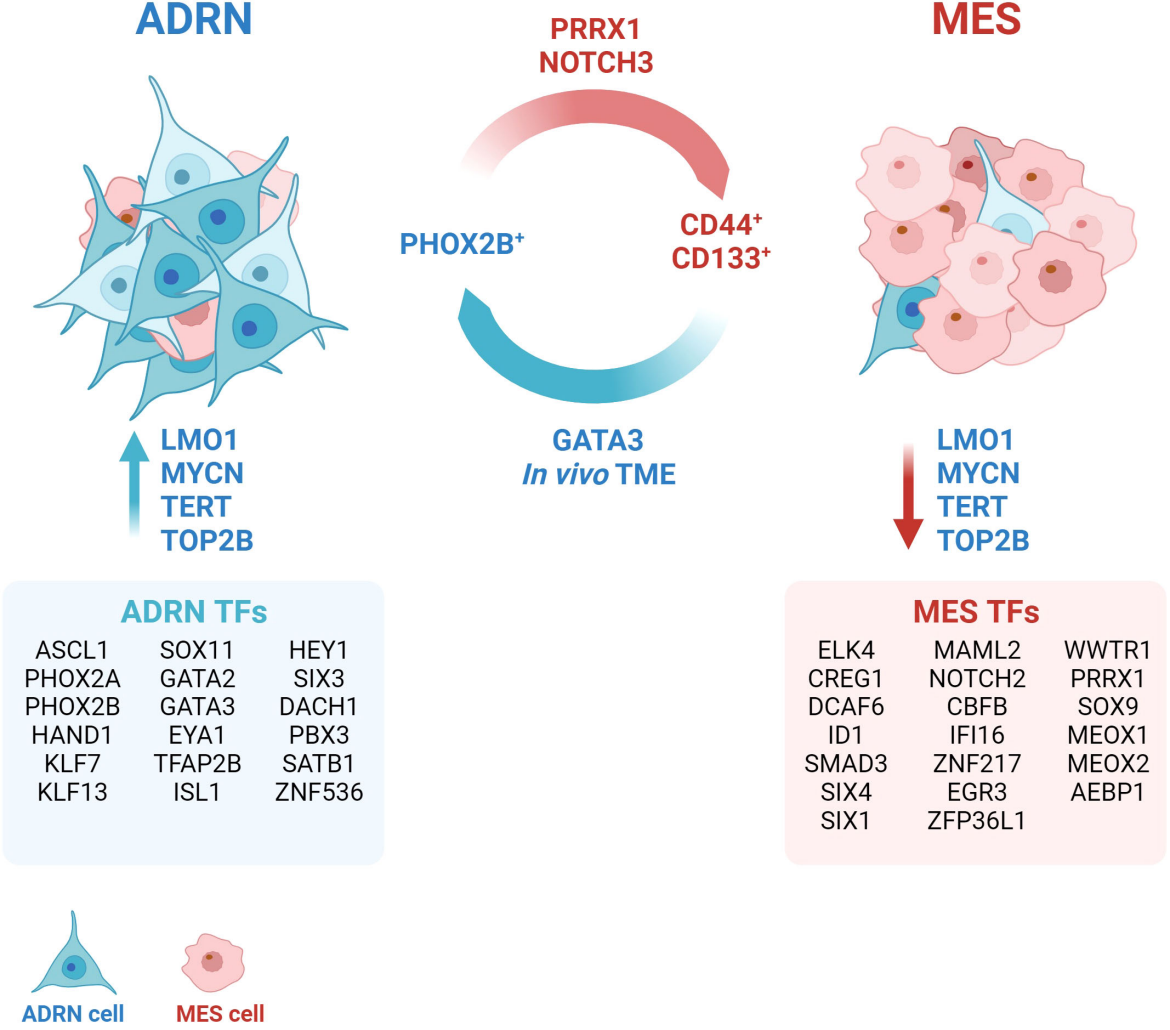
Neuroblastoma plasticity. The interconversion from the adrenergic state (ADRN) to the mesenchymal state (MES) occurs spontaneously and bidirectionally *in vivo*. The tumor microenvironment (TME) exerts a strong pressure towards the ADRN state. The transition can be induced through the activation or overexpression of specific transcription factors (TFs), such as GATA3, PRRX1, NOTCH3. The definition of cell identity is based on fundamental core regulatory circles that group different TFs (ADRN TFs and MES TFs) and establish typical gene expression signatures. Several accessory proteins, such as LMO1, TERT, TOP2B contribute to the maintenance of the transcriptional state. Created with BioRender.com.

Several studies have demonstrated that it is possible to induce ADRN/MES trans-differentiation *in vitro* by acting on lineage-specific core regulatory circuits (CRCs). In 2017, Van Groningen and colleagues showed that the over-expression of the Paired related homeobox protein 1 (PRRX1) gene, a MES-specific CRC transcription factor (TF), is able of reprogramming the transitional and epigenetic landscape of ADRN cells towards a MES state (7). Two years later, the same group described Neurogenic locus notch homolog protein 3 (NOTCH3) as a master regulator of ADRN-to-MES reprogramming (12). To date, specific CRCs have been described for ADRN and MES, consisting of 18 and 20 TFs, respectively (7) (**Figure 1**).

Many other genes have been identified to contribute to the maintenance of a specific transcriptional program. Yu and colleagues found different levels of telomeric protein expression and telomerase activity between ADRN and MES subclones (13). Accordingly, pharmacological conversion of ADRN into MES cells induced a robust change in the expression of telomere-binding proteins (13). Telomerase inhibition (TERT) was sufficient to induce a reversible switch from ADRN to MES without affecting telomere length, thus suggesting that TERT might exert a role in maintaining the ADRN phenotype independently of its telomere maintenance-related functions (13). Similarly, DNA topoisomerase 2-beta (TOP2B) was found to be required to maintain the ADRN-like transcriptional signature of SH-SY5Y cells and to suppress the alternative MES-like epigenetic state (14). Indeed, silencing of TOP2B in SH-SY5Y cells resulted in downmodulation of 47% of genes included in the ADRN signature and upregulation of 38% of genes identified in the MES signature (14). A recent study by Pan and colleagues shows that the TOP2B inhibitor CX-5461 causes DNA damage leading NB cells to apoptosis after 24 hours of treatment (15). The treatment is selective and more effective in MYCN-amplified NBs (15), in which the oncogene stabilizes the ADRN CRC (16). Inhibition of TOP2B, therefore, rather than inducing trans-differentiation from ADRN to MES, might select for the MES tumor component by specifically killing the ADRN tumor component.

Recently, a regulatory polymorphism of the LIM-domain-only 1 (*LMO1*) gene has been described to genetically determine NB fate by promoting the ADRN cell state (17). LMO1 is a transcriptional coregulator whose overexpression synergizes with MYCN to accelerate tumor formation and metastasis in a NBL-zebrafish model (18). Despite the lack of a DNA-binding domain, LMO1 mediates protein-protein interactions within ADRN CRCs and is essential for establishing ADRN cell identity (19). It has been observed that the G allele of the G → T polymorphism at the rs2168101 locus within the first intron of the *LMO1* gene predisposes to NB (20). This polymorphism, being located within a GATA domain, regulates the binding of TFs such as GATA3, and consequently affects the expression levels of LMO1. The authors showed that the protective T allele, by impairing the binding of GATA3 and reducing LMO1 expression, decreased the rate of NB initiation in MYCN-driven tumors that were restricted to the MES cell state. Whole-genome sequencing (WGS) of NB showed that tumors homozygous for the T allele (rs2168101(T;T)) have a MES cell state and are typically at low-risk NB at diagnosis (17).

It is currently unclear whether NB cells can adopt a pure MES phenotype *in vivo*, as two single-cell RNA sequencing (scRNA-seq) analyses did not identify MES-like cells in primary tumours (21, 22), and a third identified ADRN tumour cells with only a few MES-like features (23, 24). The existence of a population with similar characteristics has also been suggested on the basis of scRNA-seq analysis of 10 neuroblastic tumour samples (25). The authors described a population of “transitional cells” expressing genes involved in sympathoadrenal development, but also rapid tumor proliferation and spread, suggesting a more aggressive phenotype. In an independent cohort of NB patients, high expression of the “transitional signature” was shown to be predictive of a worse prognosis than ADRN or MES expression patterns (25). A more recent study emphasizes the intrinsic plasticity properties of NB cells and the dependence of cell identity on external signals from the environment (26). PHOX2B and CD44 have been identified as specific markers of ADRN and MES, respectively, thereby being able to separate and culture the ADRN (CD44^-^) and MES (CD44^+^) components of different cell lines with a mixed phenotype (26). The authors also observed that ADRN cells were able to acquire CD44 expression in culture over time and that this phenomenon was influenced by the composition of the culture medium. Conversely, CD44^+^ cells did not acquire PHOX2B expression, thus maintaining their MES identity *in vitro* (26). The xenografts derived from CD44^+^ and CD44^-^ cells showed a predominant ADRN identity, demonstrating the strong ability of the tumour microenvironment (TME) to drive NB cells towards ADRN differentiation. However, this is not sufficient to suppress the plasticity potential of the cells, which still retain the ability to transdifferentiate in the MES direction when isolated from the xenograft and cultured *in vitro* (26). By integrating scRNA-seq data from 18 NB biopsies and 15 patient-derived xenografts (PDXs), the authors demonstrated that human primary NB cells also acquire a predominantly ADRN phenotype (26). However, clusters of cells referred to as “bridging cells” or “noradrenergic-mesenchymal cells”, display characteristics intermediate between the two main cell identities (26). This specific cell population could be responsible of the potential plasticity of the tumour.

## 3 Cell plasticity as a driver of resistance

Dynamic and heterogeneous interconversion between tumour cell subtypes has been associated with malignant progression and responses to therapy in several cancer types, such as prostate cancer, basal cell carcinoma and lung cancer (11). Numerous efforts have been made to identify the regulatory determinants of this dynamic phenotypic plasticity and to define lineage-specific therapies (11).

A scRNA-seq analysis of parental and etoposide- or cisplatin-resistant cells shed light on the link between NB plasticity and resistance to treatment (27). This analysis showed that drug treatment induces the formation of cell subpopulations with distinct transcriptome profiles. Drug resistance was associated with the modification of drug targets and the expression of genes involved in DNA double-strand break (DSB) repair, such as BARD1, BRCA1 and PARP1 (27). Cisplatin-resistant cells were highly enriched in ADRN genes, compared to parental cells where the MES signature was prevalent. In contrast, etoposide-resistant cells were equally enriched in MES and ADRN genes, suggesting a higher plasticity potential (27).

**Figure 2:**
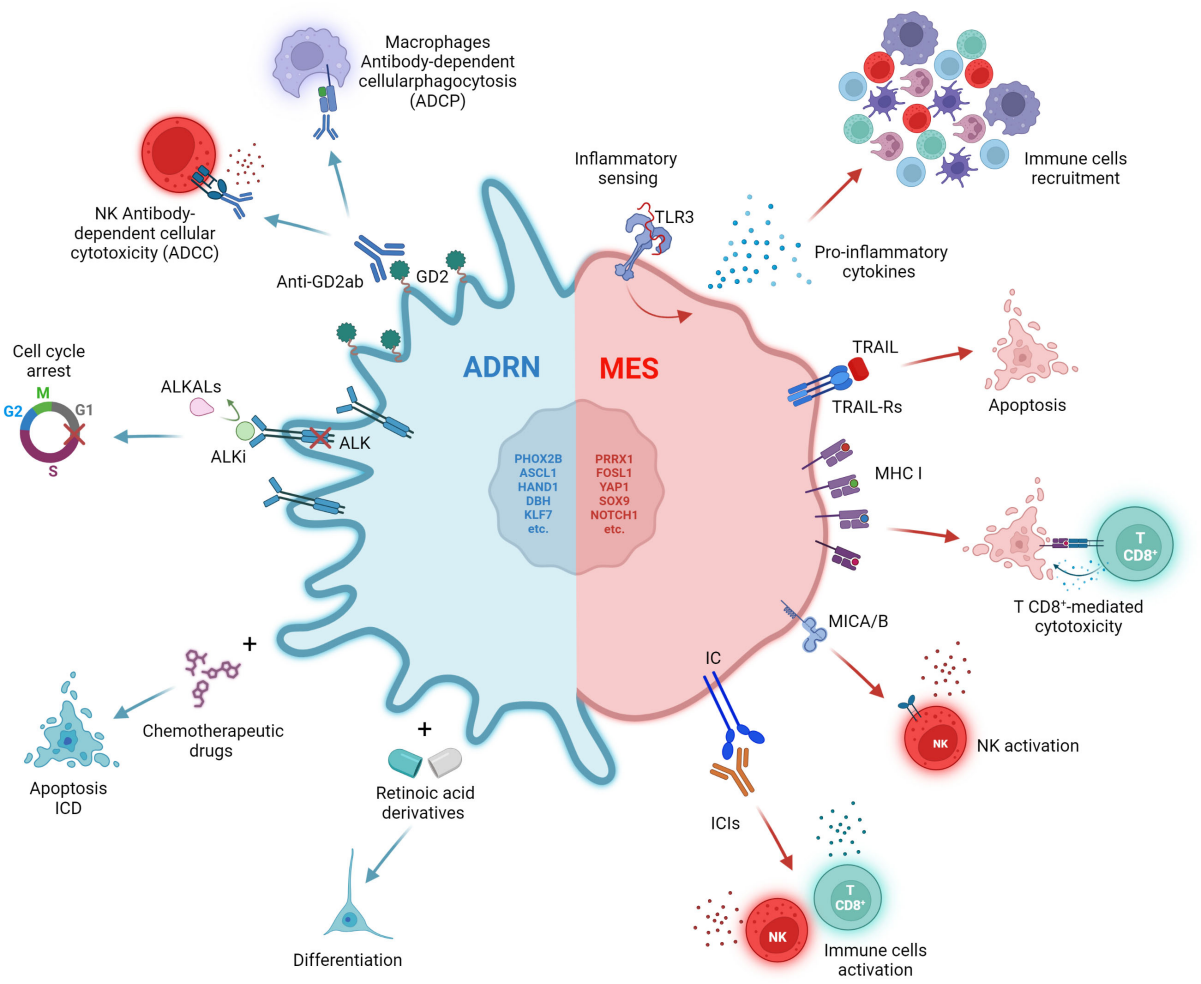
Impact of therapies on ADRN and MES states. The ADRN and MES identities are characterized by distinct vulnerabilities to therapies. The ADRN state is sensitive to differentiating and chemotherapeutic agents. The high expression of GD2 and ALK can be exploited through the use of anti-GD2 antibodies and ALK inhibitors (ALKi). The MES state displays a reduced expression of ALK and GD2, and a high immunogenicity characterized by high levels of inflammatory sensing, expression of MHC class I (MHC-I), MICA/B and immune checkpoint (IC) molecules on the cell surface, potentially emerging as a good target for ICI therapy. Created with BioRender.com.

A subsequent study isolated the ADRN and MES components of some NB cell lines and analysed their response to conventional chemotherapy, providing further evidence of how cell identity influences drug response. The authors demonstrated that MES populations show marked intrinsic resistance to conventional chemotherapy *in vitro* compared to their ADRN counterpart (26) (**Figure 2**). Furthermore, a genome-wide epigenetic profiling study of 60 NBs identified four major epigenetic subtypes driven by super-enhancer (28). Among these, the one showing higher MES characteristics was enriched in relapsed disease, suggesting a connection between the MES phenotype and recurrence (28).

The International Society of Paediatric Oncology Europe Neuroblastoma Group (SIOPEN) developed the Rapid COJEC regimen as an induction chemotherapy step (29, 30). The regimen consists of a combination of five chemotherapeutic drugs (cisplatin, carboplatin, cyclophosphamide, etoposide and vincristine) spread over three cycles, administrated alternatively in eight 10-days cycles (29, 30). Most high-risk NB patients relapse after an initial response to treatment and develop therapy-resistant tumours. To investigate the mechanisms underlying resistance to the most common therapeutic strategy in clinical practice, Mañas and colleagues developed a treatment schedule that mimics COJEC induction therapy to treat mice carrying PDXs (31). They analysed the transcriptomic and genomic changes occurring in NBs during treatment and at relapse showing that chemotherapy-resistant NBs are enriched with an immature MES-like signature resembling multipotent Schwann cell precursors, while NBs that respond Favourably to treatment show a committed ADRN phenotype similar to normal neuroblasts (31).

The acquisition of MES features may also confer resistance to differentiating agents, target therapies and anti-GD2 immunotherapy. *In vitro*, MES NB cell lines, such as SH-EP, do not undergo differentiation in response to all-trans retinoic acid (ATRA) treatment compared to their ADRN counterparts (SH-SY5Y and SK-N-BE (2)-C) (32). ATRA induces upregulation of the retinoic acid (RA) signalling markers RARA and RARB only in ADRN cell lines (32) (**Figure 2**). Accordingly, Zimmerman and colleagues demonstrated that retinoids-induced differentiation of NB cells depends on reprogramming of the adrenergic CRC and establishment of a new retino-sympathetic CRC that causes proliferative arrest and sympathetic differentiation (16). It has been reported that MES cells endogenously produce RA to promote cell motility (33), suggesting that a RA signalling pathway might be constitutively active in MES-type cells, but not associated with differentiation. Khazeem and colleagues reported similar results investigating the role of TOP2B in RA-induced gene expression and differentiation and the balance between ADRN and MES transcription programs in SH-SY5Y (14). They show that non-expression of TOP2B hinders the induction of many ATRA-response-associated genes by shifting cell identity towards a more MES phenotype. This suggests that the reduced neural differentiation stimulated by RA in TOP2B-null cells may be a result of weakened ADRN transcriptional signatures (14).

When analysing GD2 expression and publicly available RNA-seq data for 23 NB cell lines, Mabe and colleagues found that GD2-high and GD2-low expression cell lines were strongly correlated with ADRN and MES signatures, respectively (34) (**Figure 2**). Moreover, induction of ADRN to MES conversion trough overexpression of PRRX1 or NOTCH3 reduced GD2 expression and response to anti-GD2 therapy (34). They proposed that the transition from an ADRN state to a MES state reduces GD2 expression by downregulation of ST8SIA1, a gene encoding for GD3 synthase (GD3S), through the activation of EZH2, a core subunit of the polycomb repressive complex 2 (PRC2) (34).

The MES-like phenotype has been recently associated with resistance to ALK inhibitors (ALKi) due to the absence of ALK expression even in presence of tumour-driving ALK mutations, suggesting a role of ADRN-to-MES conversion in relapse (35). ALK-mutated SH-SY5Y xenografts acquire resistance to ALKi when reprogrammed by inducible expression of NOTCH3 into a MES phenotype, providing evidence that MES cells with a mutant ALK gene can escape targeted ALKi (35) (**Figure 2**). By comparing the expression profile of 8 MES and 28 ADRN cell lines, the authors discovered the differential expression of 90 apoptosis-related genes (35). In particular, several genes involved in the extrinsic apoptosis pathway were preferentially expressed in MES cells including caspase-8. Soluble recombinant human TNF-related apoptosis-inducing ligand (TRAIL), an activator of the extrinsic apoptosis pathway, was efficient in selectively inducing apoptosis in MES cells (**Figure 2**). The combination of ALKi and TRAIL delayed relapses in a subset of SH-SY5Y xenograft, demonstrating that dual targeting of both ADRN and MES could be an effective strategy in the treatment of NB (35).

Taken together, this evidence reinforces the role of cell identity and plasticity as resistance factors and supports the evidence that the MES phenotype contributes to resistance to conventional chemotherapy and the onset of relapse.

## 4 Immunotherapy to target the mesenchymal population

Although heterogeneous, NB has been widely described as a ‘cold tumor’, i.e., characterized by a lack of T-cell infiltration and therefore unable to trigger a strong immune response (36, 37). NB is characterized by a low mutational load and reduced expression of the major histocompatibility complex (MHC) class I (38, 39). An aspect that should not be underestimated is the level of inflammatory signaling in cancer cells, which has the potential to influence immune cell trafficking and recognition of cancer cells through cytokine secretion. Lower baseline inflammatory signaling has also been associated with resistance to immune checkpoint blockade (ICB) (40, 41). The study of the functional response of a group of 20 NB cell lines to different inflammatory stimuli revealed that the epigenetic state influences the inflammatory sensing and cytokines release of NB (42). All cell lines displayed a functional interferon gamma (IFNγ) signaling and except for one, showed dysfunctional detection of cytosolic DNA by cGAS-STING (42). However, heterogeneity in the detection of double-stranded RNA (dsRNA) by Toll-like receptor 3 (TLR3) and other dsRNA sensors was shown when cells treated with the dsRNA-mimetic drug poly(I:C) (42). While all non-responsive cell lines were in the ADRN state, six of the seven cell lines that showed a robust response to poly(I:C) were in the epigenetic MES state and showed increased expression of both pro-inflammatory cytokines and antigen presentation components (42) (**Figure 2**). Moreover, the forced switch of non-responsive cell lines from the ADRN state to the MES state was sufficient to restore TLR3 response signaling. A subsequent analysis of scRNA-seq data from 10 untreated high-risk NBs confirmed that tumors with stronger MES signatures had higher levels of basal inflammatory transcripts than those with stronger ADRN signatures (42).

Another study further investigated the immunological aspect of NB by analyzing RNA-seq data from 498 well-annotated primary human NB tumors and scRNA-seq data from a total of 40 tumors (43). The analytical effort led to the identification of four clusters, one of which, named C3, was found to be enriched for the expression of genes involved in immune activation and escape (43). The C3 cluster included many of the tumors with a higher MES score, again suggesting that a MES identity is associated with higher immunogenicity (43). In addition, the author showed that overexpression of PRRX1, but not its dysfunctional form, is sufficient to induce conversion of the ADRN SH-SY5Y cell line to a MES phenotype and to increase the expression of genes involved in antigen presentation and dysfunctional sensing of cytosolic DNA. Expression of MHC class I and the MICA and MICB ligands of the activating receptor NKG2D, is also increased on the cell surface (43) (**Figure 2**).

Transcriptional analysis of seven paired NB tumors, obtained at diagnosis and at relapse, confirmed the clinical relevance of this finding (43). In two cases, tumors acquired MES features at relapse, and this was accompanied by increased expression of cytotoxic T- and NK-cell signatures and immune checkpoint inhibitors PD-1 and CTLA-4 (43) (**Figure 2**). Similar data were obtained from scRNA-seq analysis of a pair of independent tumors (43). Thus, resistance or relapse associated with the transition from an ADRN to a MES phenotype is accompanied by an increase in immune cell infiltration. The expression of immune-related genes is strongly correlated with the epigenetic state of the tumor. This hypothesis was further confirmed by Cornel and colleagues who discovered the ability of the histone deacetylase inhibitor (HDACi) entinostat to increase the susceptibility of NB cells to CD8^+^ T cell- and NK cell-mediated killing (44). The effect of entinostat was mediated by increased surface expression of MHC class I and other components of the antigen processing and presentation machinery, such as TAP1, TAP2 and immunoproteasome subunits, as well as the NK activating ligands MICA and MICB. Consistent with Sengupta findings (43), this increase in immunogenicity has been correlated with a shift towards a more MES cell lineage (44).

Evidence to date suggests that immunogenicity may be the Achilles’ heel of MES cells, representing an important opportunity to be exploited to make NBs susceptible to immunotherapy. Furthermore, the association of drug resistance features with the MES phenotype is now complemented by evidence of increased vulnerability to the immune system, opening new perspectives for future therapeutic combinations.

## 5 A combinatorial approach to target ADRN and MES populations

Several studies have shown that MES cells are more resistant to conventional treatments and are enriched in post-therapy and relapse tumors (26, 28, 31). However, by analyzing the expression of ADRN and MES mRNAs in serial bone marrow samples from high-risk NB, van-Wezel found that ADRN mRNAs and MES mRNAs have distinct temporal dynamics. Specifically, ADRN mRNAs levels were high at diagnosis and during relapse, but decreased during treatment. In contrast, MES mRNAs expression increased during treatment and was associated with patients who eventually relapsed (45). Beyond differences in therapeutic response, this result suggests that MES cells still retain a great deal of plasticity and can convert to ADRN when the selective pressure exerted by therapies is removed. This implies that an effective clinical response cannot be achieved without simultaneously targeting all the different cell states that the NB can acquire, thus rendering plasticity useless as an escape mechanism. Although current treatment protocols are not designed for this purpose, some studies are moving in this direction. Consistently, it has been proposed the dual targeting of ADRN and MES components through the combination of ALKi and TNF-related apoptosis-inducing ligand (TRAIL) (35).

The prevalence of the low-immunogenic ADRN phenotype partly explains the lack of T-cell infiltration and poor response to ICIs in this tumor type. Recent studies describing the MES phenotype as more immunogenic offer the possibility to exploit this feature and improve the efficacy of ICIs. With this in mind, forcing the NB towards a more MES identity could be an interesting strategy to sensitize the tumor to immunotherapy. Cornel and colleagues have demonstrated that epigenetic drugs, such as HDACi, can reprogram NB cells towards a more MES and immunogenic phenotype, capable of stimulating killing by effector CD8^+^ T cells and NK cells (44). However, a high MES score can also be associated with higher expression of genes involved in immune evasion, such as the immune checkpoint (43). HDACi and ICIs could act synergistically on two different fronts: the former by overcoming the tumor’s poor immunogenicity through epigenetic regulation, and the latter by counteracting the immune evasion properties of the MES phenotype.

The evidence that chemotherapy-resistant NBs exhibit MES features (31), suggests that the chemotherapy itself induces a selective pressure towards the MES status. From this perspective, combining of chemotherapy and immunotherapy could represent strategy to prevent relapse, by disfavoring the establishment of a chemotherapy-resistant MES identity.

To date, the combination of immunotherapy and chemotherapy has been little explored in the treatment of pediatric cancers. It is widely believed that chemotherapy-induced immunosuppression may render immunotherapy ineffective. However, several chemotherapy drugs, including those already used in the treatment of NB, have been shown to induce immunogenic cell death (ICD) when administered at low doses (46). ICD is a particular type of cell death that can elicit immune activation by exposing the host immune system to tumor antigens and damage-associated molecular patterns (DAMPs) (47, 48) (**Figure 2**).

Based on this, we recently proposed a chemo-immunotherapy approach combining low-dose mitoxantrone (MTX) with PD-1 and TGFβ blockade (49). This combination treatment was able to induce increased expression of several chemokines involved in the recruitment of lymphoid and myeloid cell populations, such as dendritic cells (DC) and NK cells (49), which are associated with improved survival of patients with NB and other cancers (50).

## 6 Discussion

NB cells represent a dynamic entity strongly influenced by the TME and therapeutic treatments. Their plasticity underlines the ability to optimize their survival by acquiring phenotypic traits that become favorable under specific conditions. It has been shown that in both mouse and human models the TME tends to force NB cells towards a predominantly ADRN identity (26). However, analysis of pairs of tumors taken at onset and relapse has shown that a MES phenotype can be acquired during therapy (31, 43). This is associated with increased immunogenicity, suggesting the possible success of an immunotherapeutic approach against NB (43). However, analysis of MES and ADRN mRNA expression in serial bone marrow samples of high-risk NB showed an increase in MES markers only during therapy, whereas ADRN mRNAs returned to elevated levels during relapse, suggesting that the drug-induced shift from ADR to MES might be reversible (45). It has recently been suggested that an immature component of tumor cell with an intermediate phenotype between ADR and MES, termed bridging cells or noradrenergic MES cells, may be responsible for the high plasticity potential of NB (26). In a landscape so variable and susceptible to external influences, targeting the tumor based on its current condition may not be a winning strategy. Future therapeutic approaches must consider the different transcriptional programs that might be undertaken by the tumor to simultaneously target different cell identities and reduce the tumor’s chances of escaping cell death. Combinatorial therapies including an immunotherapeutic approach could be a viable strategy to exploit the immunogenicity of the chemo-resistant MES-like population and at the same time target the prominent ADRN compartment. Several combinatorial approaches have been proposed, among which metronomic chemotherapy combined with ICIs could present several advantages. Firstly, the possibility of using drugs that have already been tested in NB patients, counteracting their side effects through dose de-escalation. Furthermore, several cytotoxic drugs have demonstrated the ability to induce ICD when used at low doses; this could counteract the “cold” TME typical of NB and contribute to the enhancement of the effect of ICIs, as observed in previous studies (51, 52). In this regard, we have shown that low-dose MTX combined with immunotherapy restricts NB growth, leading to substantial tumor regression by remodeling the TME (49). Equally important is the fact that the use of metronomic chemotherapy allows treatment to be prolonged over time (53), thus maintaining selective pressure on the ADRN component, while simultaneously targeting the chemoresistant compartment through immunotherapy. Finally, DAMPs released from dying ADRN cells could trigger the release of pro-inflammatory cytokines from the MES counterpart, further enhancing the effect of combinatorial therapy.

Given the great plasticity of NB cells, a targeted approach to ADRN/MES duality may not be sufficient for a lasting therapeutic response. Regardless of the most promising therapeutic approach, new studies are needed to better understand the different transcriptional configurations that NB cells can acquire.

## Author contributions

SD’A: Conceptualization, Writing – original draft, Writing – review & editing. PT: Writing – review & editing, Investigation. PG: Writing – review & editing, Data curation. KK: Writing – review & editing, Data curation. OM: Writing – review & editing, Data curation. MD: Writing – review & editing, Data curation. FL: Writing – review & editing, Supervision. DF: Funding acquisition, Writing – original draft, Writing – review & editing, Conceptualization.
